# Reclassifying Menopausal Breast Cancer and Assessing Non-Genetic Risk Factors in Ghanaian Women: Insights from a Cohort Study

**DOI:** 10.3390/cancers17213468

**Published:** 2025-10-29

**Authors:** Claudia Adzo Anyigba, Victor Ayinbora Azusiyine, Courage Siame, Aniefiok John Udoakang, Emmanuel Lante Lamptey, Christiana Dufie Asamoah, Helena Frempong, Gordon Akanzuwine Awandare, Josephine Nsaful, Joe Nat Clegg-Lamptey, Florence Dedey, Lawrence Edusei, Ralph Armah, Alfred Twumasi, Ronald J. Weigel, Lily Paemka

**Affiliations:** 1 West African Centre for Cell Biology of Infectious Pathogens, University of Ghana, Accra P.O. Box LG 54, Ghana; 2 Department of Biochemistry, Cell and Molecular Biology, University of Ghana, Accra P.O. Box LG 54, Ghana; 3 Department of Biochemistry and Forensic Sciences, C. K. Tedam University of Technology and Applied Sciences, Navrongo P.O. Box 24, Ghana; 4 Department of Medical Biochemistry, University of Ghana Medical School, Accra P.O. Box GP 4236, Ghana; 5 Department of Biosciences and Biotechnology, University of Medical Sciences, Laje Road, Ondo City PMB 536, Nigeria; 6 Centre for Molecular Biosciences and Medical Genomics, University of Medical Sciences, Laje Road, Ondo City PMB 536, Nigeria; 7 West African Genetic Medicine Centre, University of Ghana, Accra P.O. Box LG 25, Ghana; 8 Department of Surgery, Korle Bu Teaching Hospital, University of Ghana Medical School, Accra P.O. Box 77, Ghana; 9 Department of Pathology, Korle Bu Teaching Hospital, University of Ghana Medical School, Accra P.O. Box 77, Ghana; 10 Department of Surgery, Greater Accra Regional Hospital, Accra P.O. Box GP 473, Ghana; 11 Department of Surgery, Carver College of Medicine, 1500 John Colloton Pavillion, 200 Hawkins Drive, Iowa City, IA 52242, USA

**Keywords:** breast cancer, Ghanaian, risk factors, early-onset breast cancer, premenopausal, late-onset, postmenopausal, association, reproductive factors

## Abstract

Background/Objectives: Breast cancer incidence is increasing in younger Ghanaian women. However, few epidemiological studies have evaluated the modifiable risk factors in this population. Additionally, these studies have classified breast cancer in Ghanaian women based on the global menopausal case classification. This study reclassified premenopausal and postmenopausal breast cancer in a Ghanaian cohort, assessing the risk factors using the observed menopausal age in Ghanaian women of 48 years, rather than the global standard of 50 years. Methods: Women diagnosed with breast cancer and scheduled for surgery from December 2018 to March 2023 were recruited across four hospitals in Ghana for the Ghana Breast Cancer Omics Project (BCOPGh), and data were collected using a questionnaire. Cross-tabulation and linear regression were used to evaluate the relationships between categorical variables and age at diagnosis. Results: Out of a total of 262 women recruited, 34.4% were classified as having premenopausal breast cancer, while early-onset breast cancer (EOBC) accounted for 14.9% of all cases. The molecular subtypes were predominantly hormone receptor (HR)-positive (61%) while triple-negative breast cancer (TNBC) accounted for 16%. The tumours were predominantly at stage II (62%) and grade 2 (51%), with invasive carcinoma NST (56%) being the most common subtype. Within this cohort, nulliparity increased the odds of EOBC by 13.5-fold, while having a first birth after the age of 23 doubled the odds of premenopausal breast cancer. Reproductive factors (menarche and menopause) and lifestyle (alcohol intake, smoking, contraceptive use, and breastfeeding duration) were not associated with premenopausal breast cancer in this cohort. About 13% of participants reported a family history of breast cancer, and 79% had prior knowledge of the disease. Conclusion: This study supports previous reports of the relatively higher incidence of aggressive disease in young Ghanaian women and the protective effect of early age at first birth. It further underscores the need to investigate its genetic underpinnings, whilst highlighting the importance of public education on self-examination techniques to reduce advanced disease presentation in Ghanaian women.

## Simple Summary

Breast cancer is a multifactorial disease, with racial variations often observed in its presentation. These variations are influenced by population-specific risk factors and reproductive health characteristics. Menopausal age is used as a basis for classifying breast cancer, with most studies using 50 years as a proxy for the global menopausal age. However, menopause tends to occur relatively earlier in Indigenous African populations. Considering the younger age structure and menopausal age in Ghanaian women, this study reclassified premenopausal and postmenopausal breast cancer within a cohort, using the average menopausal age of 48 years observed in Ghanaian women. We then compared the disease presentation between both classifications and evaluated the association between modifiable and nonmodifiable risk factors and premenopausal breast cancer. This study emphasises the potential of public education in reducing advanced breast cancer presentation in Ghanaian women and highlights the need to profile the genetic risk factors to understand its molecular basis in this population.

## 1. Introduction

According to the International Agency for Research on Cancer (IARC), breast cancer is the second most diagnosed cancer worldwide with an estimated 2.3 million new cases diagnosed in 2022 [1]. With over 665,000 associated deaths reported in 2022, it is the fourth leading cause of cancer-related mortalities in both sexes [2]. Approximately 8.6% of the newly diagnosed cases were recorded in African women. Despite the reported lower incidence (being the second least affected continent), Africa recorded the third highest (13.7%) proportion of breast cancer-related deaths [2]. About 33% of the new breast cancer cases and 27% of the deaths were recorded in women diagnosed younger than 50 years (premenopausal breast cancer), representing a significant and growing public health burden in Indigenous African populations [3]. Without early diagnosis and timely access to interventional resources, breast cancer mortality in sub-Saharan Africa is projected to surge to about a million deaths annually by 2030 [4]. In Ghana, the data shows that female breast cancer accounted for 31.4% (5026) of newly diagnosed cancer cases and 24% (2368) of all cancer-related mortalities, positioning it as the leading incident cancer and cause of cancer-related deaths in Ghanaian women [2]. Approximately 50% of the incidence and related mortalities were observed in premenopausal women. By 2030, newly diagnosed premenopausal breast cancer cases and their associated deaths are projected to increase by approximately 47% [5]. Extensive research has identified several nonmodifiable (including age, race, genetics, gender, and reproductive factors) and modifiable (including lifestyle-related factors and environmental exposure) factors believed to influence breast carcinogenesis [6,7,8,9]. Although nonmodifiable, reproductive factors including ages at menarche and menopause, parity, and gravidity have been variably associated with breast cancer across populations [10,11]. Yet, few studies have evaluated the association between breast cancer and the modifiable risk factors and reproductive health in Ghanaian women.

Breast cancer is a multifactorial disease mediated by an interplay between genomic and non-genetic factors, with racially varied presentations [12,13]. Despite its widespread prevalence, the aetiology of breast cancer remains unclear [14,15]. Several studies have identified several non-genetic factors including early menarche [16], delayed age at first full-term pregnancy and birth [17], late menopause [18,19], habits like smoking [20,21], alcohol consumption [22,23,24], contraceptive use [25,26], and body mass index [27,28] as contributing to an increased risk of developing breast cancer. Importantly, breast cancer is a hormonal disease with varied presentations at different ages of diagnosis [29]. The classification of breast cancer into premenopausal (diagnosed in women younger than 50 years) and postmenopausal (diagnosed in women at least 50 years old) breast cancer was developed primarily to identify and streamline age-associated risk factors. This classification stratifies cases using 50 years as the global average menopausal age [30,31,32], representing mostly Eurocentric and Caucasian populations. However, it does not consider the different age structures in different populations which significantly influences the proportions of premenopausal and postmenopausal breast cancer cases [33]. This may explain the inconsistent and conflicting risk assessment reports published across different populations [27,34], thus underscoring the need for case stratification that employs population-specific demographic features.

Menopausal age profiles vary across populations [35] with women of African ancestry reaching menopause relatively earlier than their White American and European counterparts [36]. Furthermore, the African population has the largest proportion of individuals younger than 16 years, making up approximately 40% of the population, compared with the global average of 25% [37]. This suggests that evaluating these risk factors in Indigenous African populations using the globally generated menopausal case stratifications may misrepresent the actual proportion of premenopausal and postmenopausal cases and their associated risk factors. In Ghana, there has been a considerable increase in the incidence of breast cancer in young women (<50 years) [38,39,40,41]. Despite evidence that 91% of the Ghanaian population is younger than 55 years [42] and the average menopausal age in Ghanaian women being 48 years [43,44], the few studies evaluating the non-genetic risk factors have still relied on the global case stratification [45,46]. The only study that attempted to reclassify premenopausal and postmenopausal cases while profiling the patterns of breast tumour presentation in Ghanaian women used the study cohort’s average menopausal age of 46.6 years [41]. Study-specific case classifications and risk assessments may not adequately represent the risk profile in the population. Thus, this study aimed to reclassify breast cancer cases using the Ghanaian population’s average age at menopause and reevaluate their associated non-genetic risk factors. The youthful age structure of the Ghanaian population provides an ideal context to investigate premenopausal breast cancer-associated risk factors.

## 2. Materials and Methods

### 2.1. Study Design and Ethical Considerations

This is a hospital-based, descriptive cross-sectional analysis of baseline data from a cohort of Ghanaian women diagnosed with breast cancer. Written informed consent was obtained from all participants prior to enrolment, ensuring adherence to the Declaration of Helsinki.

### 2.2. Participant Recruitment and Study Sites

Ghanaian women diagnosed with breast cancer and scheduled for surgery were recruited from four (4) hospitals in two (2) regions of Ghana from December 2018 to March 2023 for a larger project, the Ghana Breast Omics Project (BCOPGh). The recruitment sites were the Korle-Bu Teaching Hospital, Greater Accra Regional Hospital, Ghanuba Specialist Clinic in the Greater Accra region, and Ho Teaching Hospital in the Volta region. In this study, a Ghanaian woman was defined as a citizen by descent (born to at least one Ghanaian parent), belonging to an Indigenous Ghanaian ethnic group. The inclusion and exclusion criteria for participant recruitment are detailed below:

#### 2.2.1. Inclusion Criteria

Ghanaian women, aged at least 18 years, diagnosed with breast cancer and willing to offer informed consent.

#### 2.2.2. Exclusion Criteria

Women under 18 years old, unable/unwilling to offer informed consent and non-Ghanaians.

### 2.3. Data Collection and Analysis

After recruitment, a structured questionnaire, comprising three sections capturing key thematic areas, was administered to consenting participants. Section one was designed to obtain general demographics (age, tribe, occupation, and residence); section two captured participants’ reproductive history, medical history, and clinical presentation; while section three captured participants’ lifestyle history (smoking, alcohol intake, contraceptive use, and use of bleaching creams) and breast cancer knowledge. Data was captured using the REDcap www.redcap.ug.edu.gh (accessed on 15 January 2024) electronic data capture platform. The data were exported and analysed using R statistical software version 4.3.2. In this study, the cases were categorised using the previously reported average menopausal age (48 years) in Ghanaian women [43,44,47], as the cut-off threshold into premenopausal (<48 years old) and postmenopausal (≥48 years old) breast cancer cases. This study further grouped the cases into early-onset breast cancer (EOBC), which are cases diagnosed younger than 40 years, and late-onset breast cancer (LOBC), comprising those diagnosed 40 years and older, based on previous studies [6,38,48,49], although other studies have used 45 years [50]. Participants’ ethnicity was self-defined and coded into 14 Ghanaian ethnolinguistic groups, representing 11 of Ghana’s 16 regions. Gravidity was defined as the number of pregnancies, including full-term births, stillbirths, and abortions, while parity referred to the number of pregnancies that resulted in stillbirths or live births. Symptoms prompting hospital visits were recorded, allowing for the analysis of clinical presentation patterns. Knowledge of breast cancer was defined as an awareness of breast cancer prior to diagnosis, assessed by a “Yes” or “No” response to having heard of breast cancer before diagnosis and the ability to identify at least one symptom of the disease. A series of statistical tests were conducted to compare groups: the Mann–Whitney U test was employed for group comparisons and Fisher’s exact and Chi-square tests was used to assess associations between categorical variables. To evaluate the association between risk factors and the two age-based outcomes, we first conducted univariate logistic regressions on each original categorical predictor (menarche age, parity, gravidity, first birth age, contraceptive use, and occupation). To avoid sparse data bias (minimum events per variable ≥ 10) and improve model stability, we collapsed each multi-level predictor into a binary variable using epidemiologically established cut-points and similar directional effects in the univariate ORs. For the premenopausal (diagnosis < 48 years vs. ≥48 yrs.) and early-onset (diagnosis < 40 yrs. vs. ≥40 yrs.) groups, we then ran parsimonious multivariate logistic regressions including only the strongest predictors: occupation and age at first birth for premenopausal group, and parity and occupation for EOBC. We report adjusted odds ratios (ORs), 95% confidence intervals (CIs), *p*-values, area under the ROC curve (AUC), and the Hosmer–Lemeshow goodness-of-fit test. A two-sided *p* < 0.05 denoted statistical significance.

## 3. Results

### 3.1. General Demographics

#### 3.1.1. Participants’ Age Profiles and Case Group Incidence

**Figure 1:**
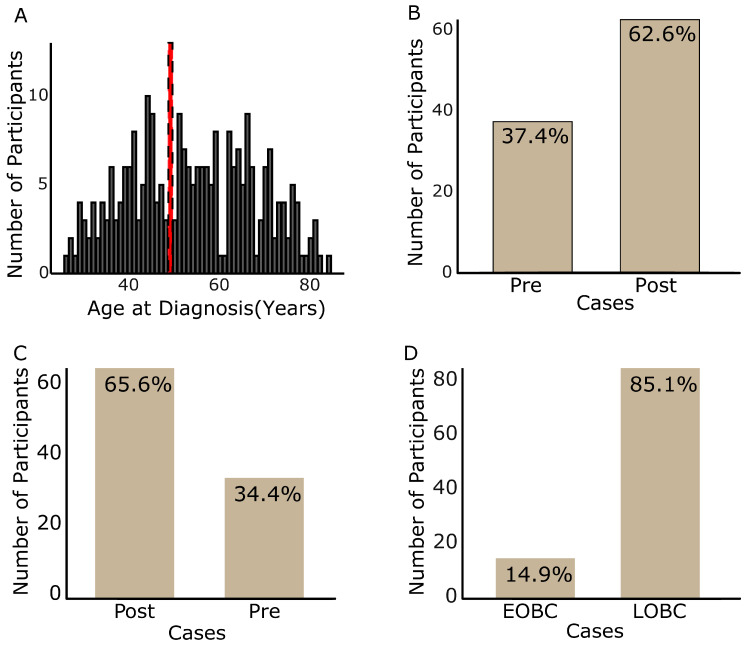
Participants’ case classification. The age range of all participants recruited (**A**), the proportion of premenopausal and postmenopausal using 50 years (**B**) and 48 years (**C**), and EOBC and LOBC (**D**) case groups. Participants’ age ranged from 27 to 83 years; the peak age at diagnosis (red dashed peaks) was 50 years (**A**). Approximately 34.4% of the cases were premenopausal breast cancer (**B**) and 14.9% were EOBC (**C**). Pre—premenopausal breast cancer; Post—postmenopausal breast cancer; EOBC—early-onset breast cancer; LOBC—late-onset breast cancer.

A total of 262 women diagnosed with breast cancer were recruited. Table 1 summarises the case groups, their reproductive factors, and general demographics. The participants were 27 to 85 years old, with a median age of 54 years. The modal age (peak breast cancer incidence age) was 50 years (Figure 1A). Notably, 37.4% of cases were categorised as premenopausal breast cancer when the cases were classified using 50 years (Figure 1B) as opposed to 34.4% using 48 years (Figure 1C), while early-onset breast cancer (EOBC) accounted for 14.9% of all the cases (Figure 1B,C). The median age at diagnosis was 35 years for EOBC and 41 years for premenopausal breast cancer (Table 1). Participants in this study were mostly traders or businesswomen (47%); 27% were students, unemployed, or retired; and 26% comprised clerical, farmers, and professional workers (Figure S1). Generally, the participants resided mostly in peri-urban (53%) and urban (44%) settings. The participants primarily belonged to the Akan (45%), Ga-Adangbe (24%), and Ewe (22%) ethnolinguistic groups, with approximately 9% comprising eight other ethnolinguistic groups (Table S1). Roughly 86% of the participants have had a mammogram. Occupation, menstrual status, and mammogram screening history showed considerable associations with the age at diagnosis.

#### 3.1.2. Participants’ Reproductive Factors

**Figure 2:**
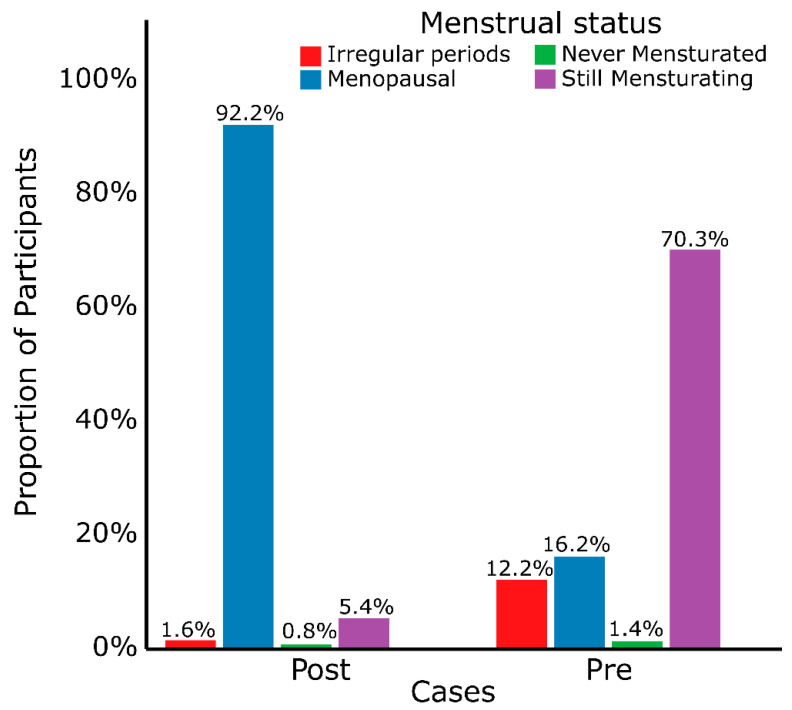
Menstrual status of participants. About 5.4% of the participants diagnosed at 48 years and older were still menstruating and 16.2% of those diagnosed below 48 years were menopausal. Two participants had never menstruated. Post—postmenopausal; Pre—premenopausal.

The median age at menopause in this study was 48 years, with the youngest and oldest menopausal participants being 27 and 60 years old, respectively (Table 1). About 65% of the participants were menopausal, 26% were still menstruating, 8.6% had irregular menstruation, and 1.4% (two women) had never menstruated (Figure 2). The median age at menopause was considerably lower (42, *p* = 0.005) in the premenopausal case group and only one EOBC participant was menopausal. The age at menarche ranged from 10 to 25 years with a median of 15 and was significantly lower (14, *p* < 0.001) in the EOBC cases. Generally, the women in this study were multiparous and multigravid, with an average of three births and four pregnancies, respectively (Table 1). Significantly lower parities were observed in participants diagnosed before the age of 48 years (2, *p* < 0.001) and were lowest in participants diagnosed at ages below 40 years (1, *p* < 0.001). Gravidity was also considerably lower in the premenopausal group (3, *p* < 0.001); this was lowest in women diagnosed younger than 40 years (2, *p* < 0.001). The overall primiparity age in this study ranged from 15 to 54 years, with a median of 24 years. This was significantly higher in the premenopausal breast cancer cases (27, *p* < 0.001).

Table 2 illustrates the association between the reproductive factors and the age at diagnosis. Notably, 9% of the women have never given birth, 32% had two children, 28% had three children, and 31% had more than three children. Approximately 17% of the participants had never been pregnant, 29% had had more than four pregnancies, and 32% had been pregnant at most three times. This study’s participants breastfed for an average of 18 months; 67% breastfed for more than 12 months, 28% for 6–12 months, 5% for at most 5 months, and 5.4% never breastfed. Overall, menopause, menarche, gravidity, parity, and primiparity correlated considerably with the age at diagnosis, whereas breastfeeding showed no association in our cohort.

### 3.2. Medical History and Clinical Presentations

Table 3 summarises the breast cancer clinicopathological characteristics of our cohort. Approximately 60.7% of the participants reported lumps in the breast and 15.6% reported painful breasts; 23.7% of the participants reported a range of symptoms including flaky skin, open wounds, discoloured breasts, and distorted breast shape (Figure S2). Approximately 68% of the participants detected these symptoms via self-examination and 32% via medical examination (Table 1). Invasive carcinoma NST, invasive ductal carcinoma, and ductal carcinoma in situ constituted 56%, 18%, and 13% of the cases, respectively; Figures S3 and S4 show the distribution of the other (6.1%) tumours. Tumour type was not associated with the symptoms presented (Figure S5) or breastfeeding duration (Figure S6). Only 47 of the participants had stage classifications and 149 had grade classifications. Approximately 60% of cases were stage II; stages I, III, and IV accounted for 19%, 15%, and 4%, respectively. Grade 2 accounted for 51% of all the cases; grades 1 and 3 constituted 21% and 28%, respectively. Hormone receptor-positive (ER+/PR+; ER+/PR-; ER-/PR+, including HER2-equivocal) accounted for 59.7% and hormone receptor-negative (ER-/PR- including HER2-equivocal), 20.6% of the cases; approximately 16% were triple-negative breast cancer (TNBC) and 4.6% were HER2-enriched. Generally, there was no association between the clinical presentations and age at diagnosis.

### 3.3. Participants’ Lifestyle and Family History

Table 4 highlights the lifestyle and family history of the participants. Approximately 21% of the participants were unaware of the disease prior to their diagnosis. Regarding habits, 99% of the participants had never smoked, 73% never drank alcohol, 13% had used skin-bleaching creams, 29% had used a contraceptive, and 13% reported having a family history of cancer. Of those who drank alcohol, 21% drink occasionally and 5% drink frequently (weekly and monthly). Contraceptive use was significantly associated with diagnosis before 40 years (*p* = 0.005).

### 3.4. Regression Analysis and Risk Assessment

Univariate analyses showed that the odds of being diagnosed at younger than 48 years significantly decreased with every unit increase in the ages at menarche and menopause, as well as the number of pregnancies (gravidity) and births (parity) (Table S2). The age at first birth increased the odds of being diagnosed before 48 years. Further categorization indicated that menarche (<15 years) decreased the odds, and zero births (parity = “never”) considerably increased the odds of being diagnosed when younger than 48 years (including EOBC). The odds of being diagnosed with EOBC were higher in women who had never been pregnant. A multivariate logistic regression analysis, summarised in Table 5, was conducted to evaluate the strongest predictors of age at diagnosis based on the results from the univariate analysis summarised in Table S3. In this study’s cohort, a reduced logistic model including occupation and first birth age showed that unemployed women had 82% lower odds of being diagnosed at younger than 48 years (OR 0.118, 95% CI 0.045–0.31, *p* < 0.001), while first birth ≥ 24 years doubled the odds (OR 2.11, 95% CI 1.15–3.86, *p* = 0.016). This model discriminated well (AUC = 0.7194) and calibrated acceptably (Hosmer–Lemeshow χ^2^ = 1.21, *p* = 0.5465). The logistic model shows that nulliparous women had 13.5-fold higher odds of diagnosis before 40 years (OR 13.49, 95% CI 5.06–35.98, *p* < 0.001), and unemployed women had 88% lower odds (OR 0.118, 95% CI 0.051–0.66, *p* = 0.009). The model’s AUC was 0.7190, and the Hosmer–Lemeshow χ^2^ = 0.74 (*p* = 0.3909), indicating good fit.

## 4. Discussion

This study aimed to more accurately define premenopausal and postmenopausal breast cancer classification and assess the non-genetic risk factors in Ghanaian women. The International Agency for Research on Cancer (IARC) ranked breast cancer as the foremost cancer diagnosed (5026, 31.4% of all new cancers) and the leading cause of cancer-related deaths (2369, 24% of newly associated deaths) in Ghanaian women [1,2]. Approximately 52% of the incident cases were observed in women younger than 50 years old (premenopausal breast cancer) and this is predicted to increase by 41% over the next decade [2]. Currently, the menopausal classification of breast cancer uses age 50 years as a proxy for the global average menopausal age [31,32]. Meanwhile, available reports indicate that menopause occurs earlier in African women than in American and European women [36]. Breast cancer is a hormonal disease with varying presentations at diagnosis [29], hence the classification into premenopausal and postmenopausal cases. The variations in menopausal age have been associated with genetic variations [51,52], socioeconomic factors [53,54,55], and lifestyle [56]. Considering the ethnic variations in breast cancer presentation and differences in age at menopause, using the global average (50 years) to evaluate breast cancer in Indigenous Africans will misrepresent their case proportions as well as prevailing risk factors. In Ghana, the few studies that have investigated the known non-genetic breast cancer risk factors have classified the cases using the global average menopausal age of 50 years [39,45], which is higher than the 48 years previously reported [43,44] in Ghanaian women and observed in this study. Ghartey et al. [41], who attempted to classify the cases, used the menopausal age (46.6 years) observed in their study cohort, which is not representative of the average menopausal age in Ghanaian women. In 2024, Bosompem et al. [47] compared the disease characteristics between premenopausal and postmenopausal breast cancer patients in Ghana. Although they recognised 48 years as the average menopausal age, their analysis focused mainly on young premenopausal women (<35 years, “YMP women”). Consequently, patients aged 35–47 years, who remain premenopausal, were misclassified as postmenopausal. Thus, this study is the first to classify breast cancer in Ghanaian women using the established national menopausal age of 48 years into premenopausal (diagnosed <48 years) and postmenopausal (diagnosed ≥48 years) breast cancers.

With this classification, 34.4% of the cases were premenopausal breast cancer, whereas this proportion increased to 37.4% using the controversial 50-year threshold—highlighting the influence of age classifications on prevalence estimation. Although both estimations are higher than the global estimate of 30.9%, they were lower than the estimated incidence in Africa (49.2%) and Asia (39%) [29]. These, however, were higher than those reported in Europe (19.9%), Oceania (32.2%), and Latin America and the Caribbean (LAC) (28.7%). In West Africa, 55.4% of the cases are reportedly premenopausal [29] and previous studies in Ghana have estimated 63.3% [41], 50% [45], 50.6% [39], and 58.9% [46]. Beyond the cut-off age used in the case classification in this study, the lower prevalences observed for both the 48- and 50-year age groups, compared to previous reports, are likely attributable to the smaller sample size and the participant recruitment criteria. This notwithstanding, the observed prevalence, compared with those reported in European and American populations, further supports evidence that Ghanaian women diagnosed with breast cancer are often younger. EOBC (participants diagnosed younger than 40 years) accounted for approximately 15% of all cases, compared with the previously reported 20.2% [38]. This observation may also be attributed to the sample size.

The observed median and modal (50 years) ages validate the decade-earlier breast cancer peak incidence reported in sub-Saharan African women, compared with their White American (62.4 years) and African American (60.8 years) counterparts [57,58,59]. Additionally, the average age (40 years) of participants diagnosed younger than 48 years (including EOBC) corroborates reports of breast cancer diagnosis in younger Ghanaian women [38,45]. More so, the median age in women diagnosed younger than 48 years (40 years) contradicts the estimated age (50 years) in low- and middle-income countries [33]. Importantly, over 80% of the Ghanaian population has been younger than 50 years for over 2 decades [37,42,60]. This may partly explain the relatively higher number of premenopausal breast cancer cases observed in Ghanaian women. However, a younger age at diagnosis is partly attributable to familial genetic predispositions [61] and environmental factors including ionising radiations [62] and air pollutants like polyaromatic hydrocarbons (PAHs) [63]. This highlights the need to explore both the genetic and environmental breast cancer risk factors specific to Ghanaian women.

Risk assessments between premenopausal and postmenopausal breast cancer cases have highlighted fundamental aetiological differences associated with reproductive health [17,57,64]. Parity and age at first birth were the only significant predictors of premenopausal breast cancer in our study, consistent with a previous report (Robertson. Primiparity after age 30 has been associated with an increased risk of postmenopausal breast cancer [17]. This study reported a median primiparity age of 24 years and a two-fold increase in the odds of premenopausal breast cancer with primiparity beyond 23 years (≥24 years). The observed median primiparity age in our study was relatively higher than the average reported in Ghanaian women (20.7 years) [65]. This may explain the observed odds of developing premenopausal breast cancer and supports evidence that early primiparity is protective against premenopausal breast cancer [66,67,68].

Multiparity is reportedly a protective factor [69]. Our study found no association between multiparity and premenopausal breast cancer, consistent with a previous report [66,70]. However, we detected markedly increased odds of EOBC, approximately 13.5-fold higher, in nulliparous women. While our findings may suggest that multiparity, irrespective of the number of births, did not influence the age at diagnosis in our study cohort, our modest sample size highlights the need for a high-powered study to better assess this association.

Similarly to previous reports [70,71,72], the median menarcheal age observed was 15 years. This is relatively higher than the estimated age in African American (12.2 years) and Caucasian American (12.9 years) [73] women, but comparable to the 14.5 years estimated in Nigerians and most SSA women [74]. Menarche was not associated with premenopausal breast cancer in this cohort, contradicting reports indicating an association of early menarche (<12 years) with breast cancer in Asia, Europe, Oceania, and North America [16]. Although this study’s age at menarche was significantly lower (14 years) among women diagnosed before age 40, it is comparable to the estimated average menarcheal age (13.82 years; 11–15 years) in Ghanaian women [70,71,72]. This may suggest that age at menarche is not a significant contributing risk factor for premenopausal breast cancer in this study, consistent with previous reports from Ghana [64], Senegal [11], and Mexico [75]. This further underscores the importance of the population-specific profiling of breast cancer risk factors.

A slight increase in breast cancer risk has been associated with increasing menopausal age after 50 years [16]. The average menopausal age in this study (48 years) was relatively lower but within the estimated range (45–51 years) in Ghanaian women [43], comparable to that reported in the Nigerian population (48 years) [76] and within the global range (45–55 years) [32]. Due to the number of participants who either forgot their ages at menopause or were still menstruating, this study could neither evaluate the association between menopausal age and age at diagnosis in this cohort nor comment on the relationship between premenopausal breast cancer and menopausal age.

Lifestyle choices including alcohol intake [22], smoking [77], and contraceptive use [25] have previously been associated with an increased risk of breast cancer. Contrary to these reports, there was no observed association between alcohol intake or duration and smoking with breast cancer in this study cohort. It is worth noting that alcohol intake and smoking among women are culturally uncommon in most Ghanaian communities, which may explain their lack of association with breast cancer in our cohort. Contrary to an earlier report [25] indicating a 7% increase in the relative risk of breast cancer in women who have ever used contraceptives, contraceptive use was not associated with premenopausal breast cancer in this study. This suggests that alcohol intake, irrespective of frequency, smoking, and contraceptive use may not be contributing risk factors in this study’s cohort. Additionally, breastfeeding duration (≤6 months) has been associated with an increased risk of breast cancer [78]. Participants in this study breastfed for 18 months on average and the duration showed no relationship with the age at diagnosis. This could explain the lack of a protective effect of breastfeeding in our population, where breastfeeding is culturally normative and nearly all women breastfeed for extended periods, similar to observations in Black South African women [79]. Additionally, potential recall bias regarding the exact duration of breastfeeding could have contributed to misclassification, diminishing the observed association. Nonetheless, this suggests that having ever breastfed or the duration of breastfeeding may not have influenced the age at diagnosis in this study’s cohort, similar to reports by Butt et al. [80]. This is contrary to previous indications of a reduced risk for at least 12 months of breastfeeding [81,82] and breastfeeding being a protective factor [83].

Consistent with previous reports, invasive carcinoma no special type (NST) was the most common breast cancer subtype in our cohort [38]. Similarly, the proportion was higher in LOBC (57%) compared with EOBC (54%) in our cohort. The observed proportion was, however, higher in the premenopausal case group (64%) compared with 51% in the postmenopausal case group; this may further highlight the impact of age classification on the disease characteristics. The proportion of ductal carcinoma in situ in our cohort was similarly higher in EOBC (16%), compared with LOBC (13%). Invasive lobular carcinoma was recorded in 2.7% and 5.1% of the EOBC and LOBC cases, respectively, comparable to previously reported frequencies in EOBC (3%) and LOBC (5%) [84]. Invasive ductal carcinoma was the second most diagnosed type in this study, contrary to previous studies reporting it as the most common [46,85]. However, this study’s overall frequency of invasive ductal carcinoma and ductal carcinoma in situ compared with others like invasive lobular carcinoma aligns with the general trend where most breast cancers are ductal carcinomas. Mucinous carcinoma is rare and constitutes 2% of breast cancer cases globally [86]. In our study, it accounted for approximately 3% of all the cases, similar to the 3.1% reported by Akakpo et al. [38]. However, contrary to observations made by Akakpo et al. [38], who recorded a 3.7% prevalence of mucinous carcinoma in EOBC cases, none were recorded in the EOBC cases in this study. Our observation may depend on the number of participants in this study (262), compared with 2418 participants in the study by Akakpo et al. Meanwhile, the proportion of mucinous carcinoma observed in the LOBC cases (3.4%) in this study was marginally higher than that reported by the same study (3.1%).

Hormone receptor (HR)-positive tumours were the most prevalent receptor subtypes in this study and were more frequent in postmenopausal cases, as previously reported [38,84]. Similarly to the report by Akakpo et al. [38], HR-negative tumours were more frequent in the cases diagnosed younger than 48 years (25%), compared with 17% in the postmenopausal cases. However, the proportion of HER2-enriched tumours recorded in this study was lower than was reported by Akakpo et al. [38]. This may be attributed to the sample size in this study and the absence of molecular subtype data for 42% of the participants. The report by Akakpo et al. [38] indicated that TNBC was the most prevalent molecular subtype in EOBC cases, accounting for 26.4% of the cases which is similar to the 25% prevalence previously reported in Indigenous African women [87]. With 12% prevalence, Bosompem et al. [47] also reported that TNBC was the most prevalent in young-onset breast cancer. In this study, TNBC accounted for 19% of all the subtypes in the EOBC cases but was not the most predominant molecular subtype, similar to reports by Andrikopoulou et al. [84]. Although our observation is partly attributable to the sample size, the TNBC frequency in this study’s EOBC cases was higher (19%) than the 12% reported by Bosompem et al. [47]. Notably, the frequency of TNBC tumours in this study’s EOBC cases is 1.2-fold higher than in the LOBC cases. This is marginally higher than the 1.1-fold higher TNBC frequency in EOBC cases (26.4%) than in LOBC cases (24%) reported by Akakpo et al. [38]. Though no relationship was observed between the tumour stage and grade, the participants presented mainly with stage II and grade 2 tumours. Our finding is inconsistent with most previous reports showing higher proportions of advanced tumours in Ghanaian women [38,41,47,88]. However, it corroborates the approximately 51% grade 2 presentation reported in West Africa [87]. Even so, this observation may also be because participants presenting with advanced disease are often immediately put on neoadjuvant therapy before surgery and may have been missed because only participants scheduled for surgery were recruited. In this study, stages III and IV were 1.8- and 1.4-fold higher, respectively, and grade 3 tumours were 1.32-fold higher in the premenopausal cases, compared with the postmenopausal cases. Although no stage IV tumour was recorded in the EOBC cases, the proportion of grade 3 tumours was 1.32-fold higher than observed in the LOBC cases, comparable to the 1.4-fold by Bosompem et al. [47] and higher than the 1.2-fold by Adrikopoulou et al. [84]. The increased grade 3 tumours and TNBC proportions in the EOBC group in this study corroborate the literature suggesting that Ghanaian EOBC cases present with more aggressive tumours [38,89,90]. Advanced disease presentations have been associated with late detection and a lack of knowledge or awareness of the disease [91]. The lower proportion of tumour stage and grade observed in this study may be due to the sample size as well as the number of participants with incomplete tumour classification data. Despite this, the observed proportions may also point to early detection in this cohort owing to the increasing awareness of the disease in Ghana. Although Ghana lacks national screening programmes, several breast cancer awareness and screening initiatives, mostly spearheaded by local non-governmental institutions, are conducted throughout the year, with activities typically amplified in October. These activities aim to overcome the myths, misconceptions, and sociocultural beliefs that have been previously linked to poor disease outcomes in the country, while encouraging and supporting early diagnosis. In our study, approximately 79% of the participants were aware of breast cancer before diagnosis, with premenopausal cases (85%) demonstrating higher awareness compared with postmenopausal cases (75%). This awareness may have contributed to the proportion of participants who detected the tumour via self-examination (68%) as opposed to medical exams (32%) and partly explains the reduced risk of EOBC associated with students and retirees in this study. Students are generally more technologically adept, with easy access to health information; retirees have greater peer experience with diseases and are more responsive to symptoms. While awareness does not stop the disease from developing, it informs lifestyle choices that might decrease their risk of developing the disease at a younger age or being diagnosed at advanced stages. This high rate of self-detection highlights the potential of early detection programmes, especially in regions with limited regular medical screening. It further underscores the need for improved public health campaigns focused on self-examination techniques in this population.

Overall, participants in this study reported a 13% family history of cancer, the highest recorded in Ghana to the best of our knowledge. About 10% of women diagnosed with EOBC reported a known family history of breast cancer. This is higher than the previous 6.5% reported by Ahearn et al. [92] but lower than 12.9%, which was reported by Bosompem et al. [47]. This may be attributed to the increasing knowledge of the disease. Increasing knowledge on breast cancer encourages dialogue among relatives which can reveal the disease pattern in the family. Meanwhile, the increasing familial history of cancer may also account for the younger age at diagnosis in Ghanaians. Breast cancer is a complex genetic disease; the younger age at onset and the proportion of familial cancer history make this population ideal for investigating potentially novel genetic breast cancer risk factors.

The findings of this study should be interpreted with a few limitations in mind. This cross-sectional study’s selection criteria only recruited breast cancer patients scheduled for surgery, potentially excluding those undergoing other forms of therapy at the time of recruitment. This may have inadvertently biassed the proportion of the cases. Additionally, this study relied on clinical data available at the point of participant recruitment. There was missing clinical information that could not be retrieved due to the pathology centres’ varied participant identification systems for tumour diagnosis. Finally, information beyond the clinicopathological data were provided by the participants, some of whom were unable to recollect pertinent reproductive health details including ages at menarche, first birth, menarche, and menopause. This may have also influenced the results of the risk assessment.

## 5. Conclusions

Premenopausal and postmenopausal breast cancer was reclassified using 48 years, the menopause age reported in the Ghanaian population. Approximately 34.4% of the participants were classified as having been diagnosed with premenopausal breast cancer, as opposed to 37.4% using the controversial 50-year threshold. In this study, age at first birth above 23 years and nulliparity emerged as the only reproductive factors examined in this study that independently influenced the odds of age at diagnosis. Furthermore, this study confirms the reported high prevalence and aggressive biology of premenopausal breast cancer in Ghanaian women. Additionally, this study suggests the potential of public health campaigns focused on self-examination techniques in early detection and possibly reducing advanced breast cancer presentations in Ghanaian women. Furthermore, it records the highest reported family history of breast cancer in Ghanaian women. Breast cancer is a complex genetic disease with racial variations, and the African population, being the most genetically diverse, presents a unique opportunity to identify novel breast cancer genes and gene variants specific to Indigenous African populations. The paucity of genetic data on the Ghanaian population, combined with the proportions of familial history recorded in this study underscores the need to investigate both germline and somatic genetic risk factors in Ghanaian women.

## Figures and Tables

**Table 1:** General demographics.

		Menopausal Description				Onset Description		
Characteristic	N	Overall N = 262 ^1^	Post N = 172 ^1^	Pre N = 90 ^1^	*p*-Value ^2^	EOBC N = 39 ^1^	LOBC N = 223 ^1^	*p*-Value ^3^
Age at Diagnosis	262	54 (27, 85)	63 (48, 85)	41 (27, 47)		35 (27, 39)	57 (40, 85)	
Menopause age	120	48 (27, 60)	49 (32, 60)	42 (27, 45)	<0.001	27 (27, 27)	48 (32, 60)	0.087
Menarche age	192	15 (10, 25)	15 (10, 25)	15 (11, 19)	0.005	14 (11, 17)	15 (10, 25)	<0.001
Primiparity age	233	24 (15, 54)	23 (15, 54)	27 (15, 41)	<0.001	27 (16, 35)	24 (15, 54)	0.024
Parity	259	3 (0, 9)	3 (0, 9)	2 (0, 7)	<0.001	1 (0, 4)	3 (0, 9)	<0.001
Gravidity	232	4 (0, 10)	4 (0, 10)	3 (0, 10)	<0.001	2 (0, 5)	4 (0, 10)	<0.001
**Tribe**	246				0.10			0.070
Akan		110 (45%)	74 (45%)	36 (44%)		16 (50%)	94 (44%)	
Ewe		53 (22%)	29 (18%)	24 (29%)		11 (34%)	42 (20%)	
Ga-Adangbe		60 (24%)	46 (28%)	14 (17%)		3 (9.4%)	57 (27%)	
Others		23 (9%)	15 (9.1%)	8 (9.8%)		2 (6.3%)	21 (9.8%)	
Menstrual status	262				<0.001			<0.001
Postmenopausal		171 (65%)	155 (90%)	16 (18%)		2 (5.1%)	169 (76%)	
Premenopausal		67 (26%)	7 (4%)	60 (67%)		29 (74%)	38 (17%)	
others		24 (9%)	10 (6%)	14 (16%)		8 (21%)	16 (7.2%)	
**Occupation**	262				<0.001			<0.001
Others		67 (26%)	31 (18%)	36 (40%)		18 (46%)	49 (22%)	
Student/Unemployed/Retired		72 (27%)	67 (39%)	5 (5.6%)		3 (8%)	69 (31%)	
Trader/Businesswoman		123 (47%)	74 (43%)	49 (54%)		18 (46%)	105 (47%)	
Residence	252				0.2			0.7
Peri-Urban		133 (53%)	94 (57%)	39 (45%)		19 (51%)	114 (53%)	
Rural		7 (3%)	5 (3%)	2 (2%)		0 (0%)	7 (3.3%)	
Urban		112 (44%)	66 (40%)	46 (53%)		18 (49%)	94 (44%)	
Detection	201				0.3			0.2
ME		64 (32%)	39 (29%)	25 (37%)		6 (22%)	58 (33%)	
SE		137 (68%)	94 (71%)	43 (63%)		21 (78%)	116 (67%)	
Mammogram	253				0.009			<0.001
No		35 (14%)	16 (10%)	19 (22%)		12 (32%)	23 (11%)	
Yes		218 (86%)	149 (90%)	69 (78%)		26 (68%)	192 (89%)	

^1^ N (%) for categorical; median (range) for continuous. ^2,3^ Wilcoxon’s rank sum test; Pearson’s Chi-squared test; Fisher’s exact test. ME—medical examination; SE—self-examination; Pre—premenopausal; Post—postmenopausal.

**Table 2:** Participants’ reproductive factors.

	Menopausal Description					Onset Description		
Reproductive Factors	N	Overall N = 262 ^1^	Post N = 172 ^1^	Pre N = 90 ^1^	*p*-Value ^2^	EOBC N = 39 ^1^	LOBC N = 223 ^1^	*p*-Value ^2^
Primiparity	233				0.002			0.10
24		14 (6%)	12 (8%)	2 (2.7%)		0 (0%)	14 (6.7%)	
>24		109 (47%)	62 (39%)	47 (64%)		16 (67%)	93 (44%)	
<24		110 (47%)	85 (53%)	25 (34%)		8 (33%)	102 (49%)	
Menarche	192				0.012			0.002
15		48 (25%)	27 (23%)	21 (28%)		10 (33%)	38 (23%)	
>15		66 (34%)	50 (42%)	16 (22%)		2 (6.7%)	64 (40%)	
<15		78 (41%)	41 (35%)	37 (50%)		18 (60%)	60 (37%)	
Menopausal age	120				<0.001			0.5
48		11 (9%)	11 (10%)	0 (0%)		0 (0%)	11 (9.2%)	
>48		55 (46%)	55 (50%)	0 (0%)		0 (0%)	55 (46%)	
<48		54 (45%)	44 (40%)	10 (100%)		1 (100%)	53 (45%)	
Gravidity	262				0.018			<0.001
0		44 (17%)	24 (14%)	20 (22%)		15 (38%)	29 (13%)	
4		58 (22%)	40 (23%)	18 (20%)		5 (13%)	53 (24%)	
>4		77 (29%)	60 (35%)	17 (19%)		4 (10%)	73 (33%)	
<4		83 (32%)	48 (28%)	35 (39%)		15 (38%)	68 (30%)	
Parity	262				<0.001			<0.001
0		23 (9%)	6 (4%)	17 (19%)		14 (36%)	9 (4%)	
3		73 (28%)	49 (28%)	24 (27%)		7 (18%)	66 (30%)	
>3		82 (31%)	64 (37%)	18 (20%)		3 (7.7%)	79 (35%)	
<3		84 (32%)	53 (31%)	31 (34%)		15 (38%)	69 (31%)	
Breastfeed	242				0.5			0.14
No		12 (5%)	7 (4%)	5 (6%)		3 (11%)	9 (4.2%)	
Yes		230 (95%)	157 (96%)	73 (94%)		24 (89%)	206 (96%)	
Breastfeeding duration	222				0.11			0.6
6–12		63 (28%)	48 (31%)	15 (22%)		5 (24%)	58 (29%)	
>12		149 (67%)	97 (63%)	52 (76%)		16 (76%)	133 (66%)	
<6		10 (5%)	9 (6%)	1 (2%)		0 (0%)	10 (5%)	

^1^ N (%) for categorical variables. ^2^ Fisher’s exact test; Pearson’s Chi-squared test. Pre—premenopausal; Post—postmenopausal, EOBC—early-onset breast cancer; LOBC—late-onset breast cancer.

**Table 3:** Clinicopathological characteristics of participants’ tumours.

		Menopausal Description				Onset Description		
Characteristics	N	Overall N = 262 ^1^	Post N = 172 ^1^	Pre N = 90 ^1^	*p*-Value ^2^	EOBC N = 39 ^1^	LOBC N = 223 ^1^	*p*-Value ^2^
Tumours	212				0.5			>0.9
Carcinoma NST		2 (1%)	1 (1%)	1 (1.3%)		0 (0%)	2 (1%)	
Ductal Carcinoma in situ		28 (13%)	19 (14%)	9 (12%)		6 (16%)	22 (13%)	
Inflammatory Carcinoma		2 (1%)	2 (2%)	0 (0%)		0 (0%)	2 (1%)	
Invasive Carcinoma NST		119 (56%)	67 (51%)	52 (64%)		20 (54%)	99 (57%)	
Invasive Ductal Carcinoma		38 (18%)	24 (18%)	14 (18%)		8 (22%)	30 (17%)	
Invasive Lobular Carcinoma		10 (5%)	9 (7%)	1 (1.3%)		1 (3%)	9 (5%)	
Other Malignant		13 (6%)	9 (7%)	4 (5.2%)		2 (5%)	11 (6%)	
Tumour Stage	47				0.5			0.8
I		9 (19%)	4 (15%)	5 (25%)		3 (27%)	6 (17%)	
II		29 (62%)	19 (70%)	10 (50%)		6 (55%)	23 (64%)	
III		7 (15%)	3 (11%)	4 (20%)		2 (18%)	5 (14%)	
IV		2 (4%)	1 (3.7%)	1 (5.0%)		0 (0%)	2 (5.6%)	
Tumour Grade	149				0.4			0.7
1		31 (21%)	22 (24%)	9 (16%)		5 (19%)	26 (21%)	
2		76 (51%)	46 (51%)	30 (52%)		12 (46%)	64 (52%)	
3		42 (28%)	23 (25%)	19 (33%)		9 (35%)	33 (27%)	
Molecular Classification	152				0.7			0.9
HER2-enriched		4 (3%)	3 (3%)	1 (1.8%)		1 (3.8%)	3 (2.4%)	
HR-		29 (19%)	15 (16%)	14 (25%)		6 (23%)	23 (18%)	
HR-_HER2_equivocal		1 (1%)	1 (1%)	0 (0%)		0 (0%)	1 (0.8%)	
HR+		85 (56%)	56 (58%)	29 (52%)		13 (50%)	72 (57%)	
HR+_HER2_equivocal		8 (5%)	6 (6%)	2 (3.6%)		1 (3.8%)	7 (5.6%)	
TNBC		25 (16%)	15 (16%)	10 (18%)		5 (19%)	20 (16%)	
Symptoms	262				0.5			0.14
Lump in breast		159 (60.7%)	101 (59%)	58 (64%)		29 (74%)	130 (58%)	
Others		62 (23.7%)	41 (24%)	21 (23%)		7 (18%)	55 (25%)	
Painful breast		41 (15.6%)	30 (17%)	11 (12%)		3 (7.7%)	38 (17%)	

^1^ N(%). ^2^ Fisher’s exact test; Pearson’s Chi-squared test. HR—hormone receptor, TNBC—triple-negative breast cancer; NST—no special type; Pre—premenopausal; Post—postmenopausal, EOBC—early-onset breast cancer; LOBC—late-onset breast cancer.

**Table 4:** Participant’s family history and lifestyle.

	Menopausal Description					Onset Description		
Characteristic	N	Overall N = 262 ^1^	Post N = 172 ^1^	Pre N = 90 ^1^	*p*-Value ^2^	EOBC N = 39 ^1^	LOBC N = 223 ^1^	*p*-Value ^2^
Contraceptives	244				0.3			0.005
No		174 (71%)	116 (73%)	58 (67%)		18 (51%)	156 (75%)	
Yes		70 (29%)	42 (27%)	28 (33%)		17 (49%)	53 (25%)	
Alcohol	242				0.8			0.9
Daily		3 (1%)	3 (2%)	0 (0%)		0 (0%)	3 (1%)	
Monthly		9 (4%)	6 (4%)	3 (3%)		1 (3%)	8 (3%)	
Not at all		177 (73%)	113 (73%)	64 (73%)		30 (81%)	147 (72%)	
Weekly		2 (1%)	1 (1%)	1 (1%)		0 (0%)	2 (1%)	
Yearly		51 (21%)	31 (20%)	20 (23%)		6 (16%)	45 (22%)	
Smoking	251				0.5			>0.9
No		249 (99%)	160 (99%)	89 (100%)		38 (100%)	211 (99%)	
Yes		2 (0.8%)	2 (1%)	0 (0%)		0 (0%)	2 (0.9%)	
Bleaching cream	262				0.070			0.3
No		228 (87%)	145 (84%)	83 (92%)		36 (92%)	192 (86%)	
Yes		34 (13%)	27 (16%)	7 (7.8%)		3 (7.7%)	31 (14%)	
Family History	262				0.7			0.5
No		227 (87%)	150 (87%)	77 (86%)		35 (90%)	192 (86%)	
Yes		35 (13%)	22 (13%)	13 (14%)		4 (10%)	31 (14%)	
Knowledge	242				0.10			0.8
Yes		190 (79%)	119 (75%)	71 (85%)		27 (77%)	163 (79%)	
No		52 (21%)	39 (25%)	13 (15%)		8 (23%)	44 (21%)	

^1^ N (%) for categorical. ^2^ Pearson’s Chi-squared test; Fisher’s exact test. Pre—premenopausal; Post—postmenopausal, EOBC—early-onset breast cancer; LOBC—late-onset breast cancer.

**Table 5:** Multivariate logistic regression analysis.

	Premenopausal		EOBC	
Predictor	OR ^a^ (95%CI)	*p*-Value	OR ^a^ (95% CI)	*p*-Value
Occupation				
Employed/Self-Employed	__	__	__	__
Unemployed	0.118 (0.045–0.310)	<0.001	0.183 (0.051–0.656)	0.009
Primiparity				
<24	__	__	__	__
≥24	2.108 (1.151–3.859)	0.016	__	__
Parity				
Parous	__	__	__	__
Nulliparous	__	__	13.491 (5.059–35.980)	<0.001

^a^ Adjusted odds ratio, CI—confidence interval. Model performance: premenopausal group: AUC = 0.7194; Hosmer–Lemeshow χ^2^ = 1.21; *p* = 0.547. Model performance: EOBC (N = 262): AUC = 0.7190; Hosmer–Lemeshow χ^2^ = 0.74; *p* = 0.3909. Model 1 (premenopausal) included occupation and age at first birth. Model 2 (EOBC) included parity and occupation. Employed/self-employed included traders, business women, farmers, secretary/clerical workers, and professional workers; student/unemployed consisted of students and retirees. Model performance: premenopausal (N = 233): AUC = 0.7194; Hosmer–Lemeshow χ^2^ = 1.21; *p* = 0.547. EOBC—early-onset breast cancer.

## Data Availability

The data presented in this study are available on request from the corresponding author due to privacy.

## Supplementary Materials

The following supporting information can be downloaded at: https://www.mdpi.com/article/10.3390/cancers17213468/s1, Figure S1: Occupation of the participants; Figure S2: Range of symptoms reported by the participants; Figure S3: Breast cancer subtypes in the EOBC and LOBC groups; Figure S4: Breast cancer subtypes in premenopausal and postmenopausal breast cancer groups; Figure S5: Relationship between the tumour subtypes and symptoms; Figure S6: Relationship between breastfeeding duration and tumour subtypes; Figure S7: Proportion of pre- and postmenopausal breast cancer categorized using 50 years. Pre—premenopausal; Post—postmenopausal; Table S1: Regional origin of the ethnolinguistic groups of the participants; Table S2: Logistic regression; Table S3: Univariate analysis.
