# Supplementary material for: Reclassifying Menopausal Breast Cancer and Assessing Non-Genetic Risk Factors in Ghanaian Women: Insights from a Cohort Study
Source: Cancers (Basel). 2025 Oct 29;17(21):3468. doi: 10.3390/cancers17213468 (PMC12608925; doi:10.3390/cancers17213468)
Supplement: Supplementary file 1 [file cancers-17-03468-s001.zip › cancers-3878412-supplementary.pdf]

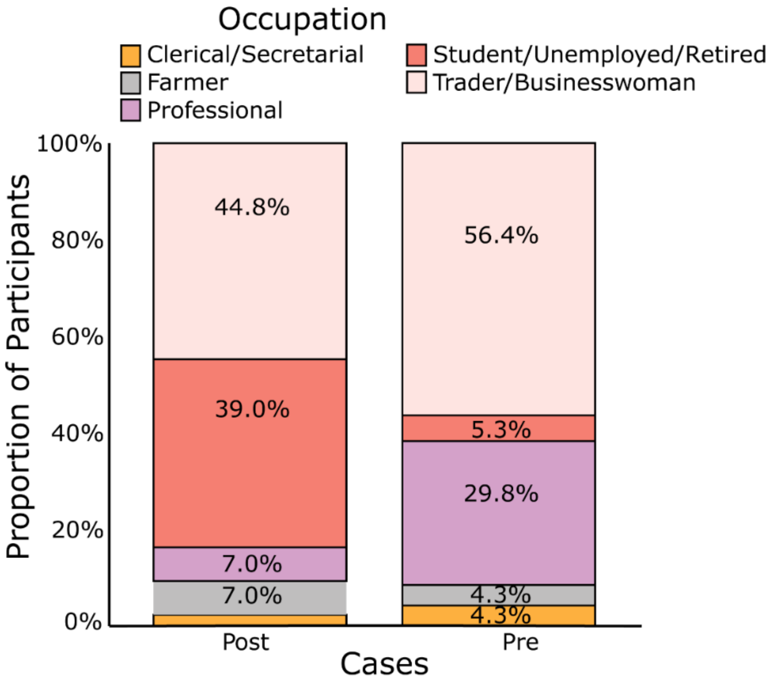

Figure S1: Participants' Occupation. The Majority of the participants were traders or Businesswomen. In the postmenopausal group, 39% were unemployed and retired, while clerical workers or secretaries represented the smallest proportion (2.3%). Approximately 30% of the premenopausal group had professional jobs, and the least represented occupations were farmers and students/unemployed. Post – postmenopausal; Pre - premenopausal

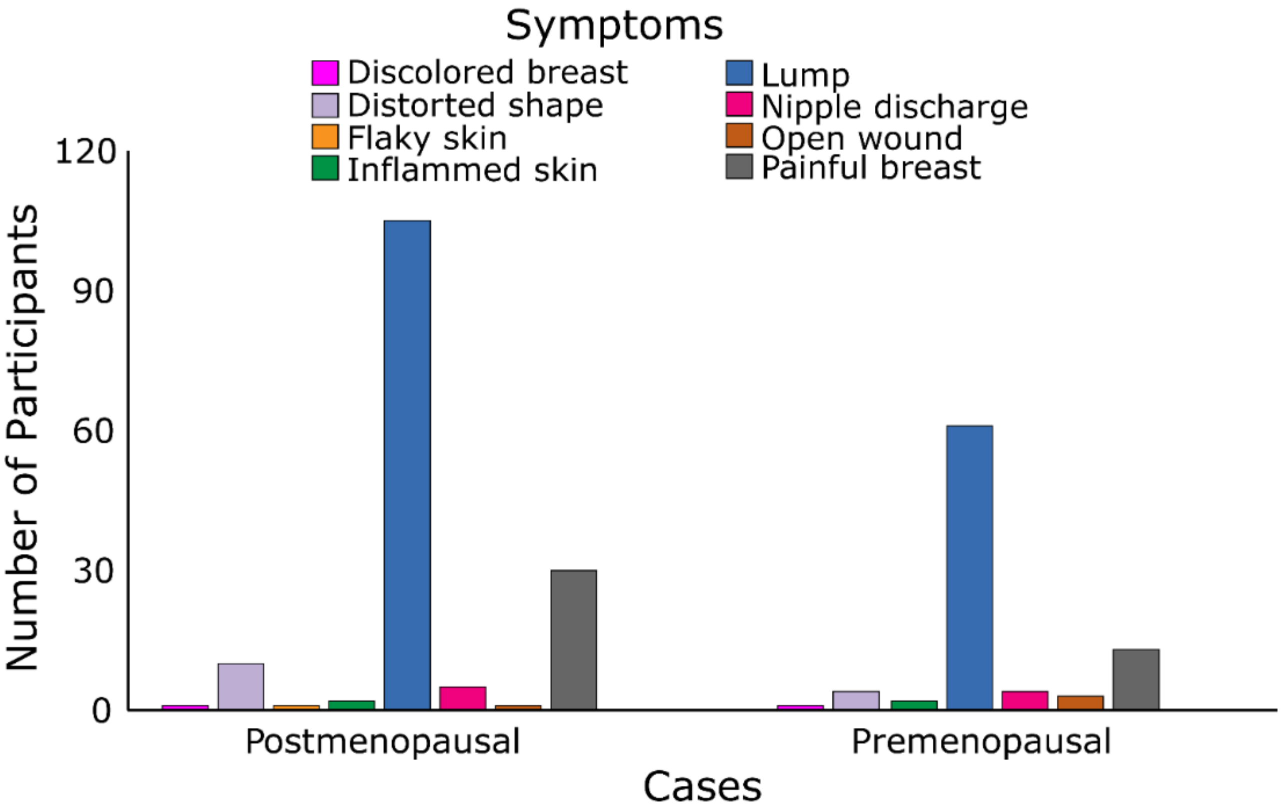

Figure S2: Range of symptoms reported by the participants. The most common symptom reported was a lump in the breast followed by painful breasts. One patient aged >50 years was presented with flaky skin.

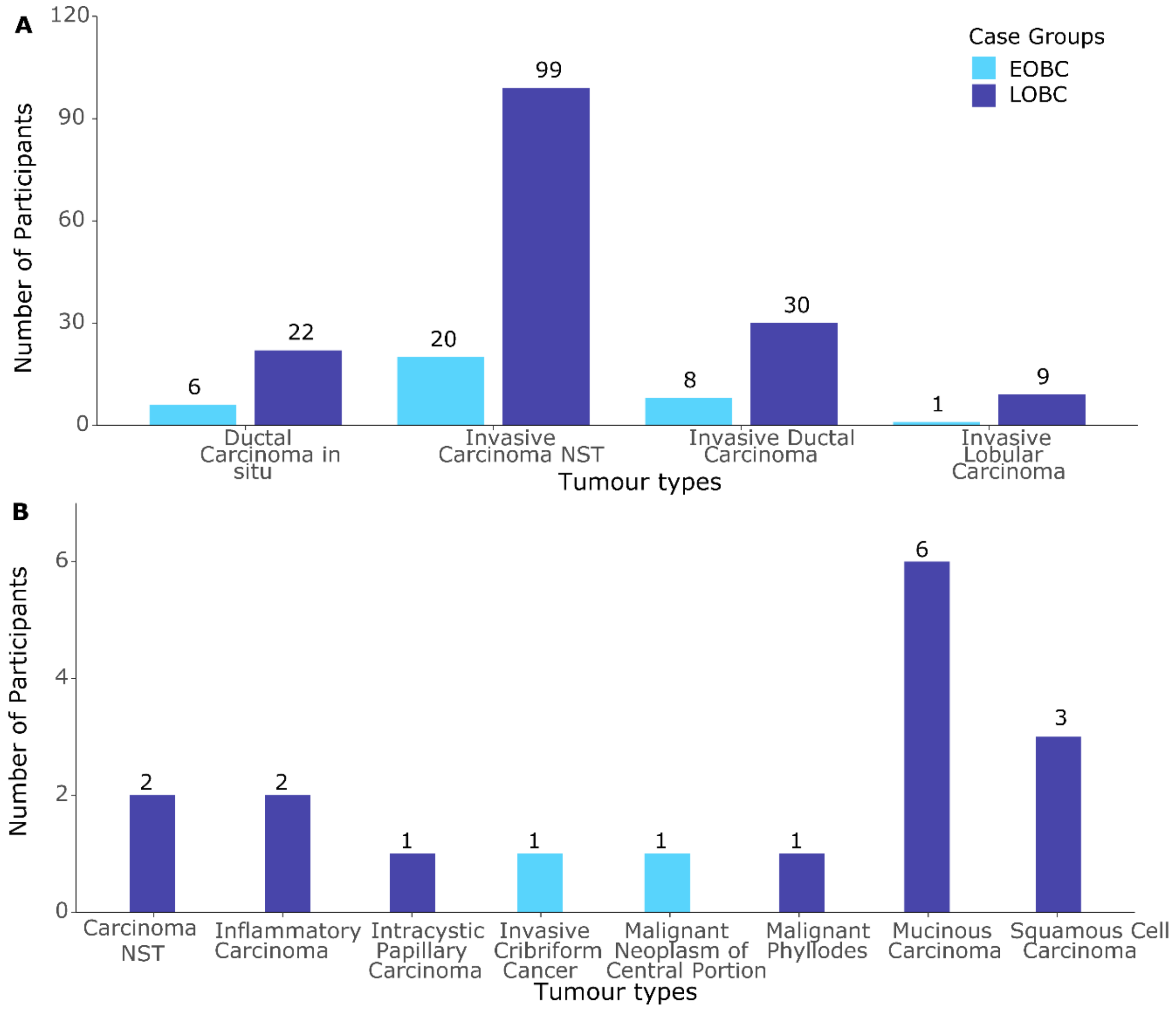

Figure S3: Breast cancer subtypes in the EOBC and LOBC cases. Tumour types present in both groups (A) and exclusive to each group (B).

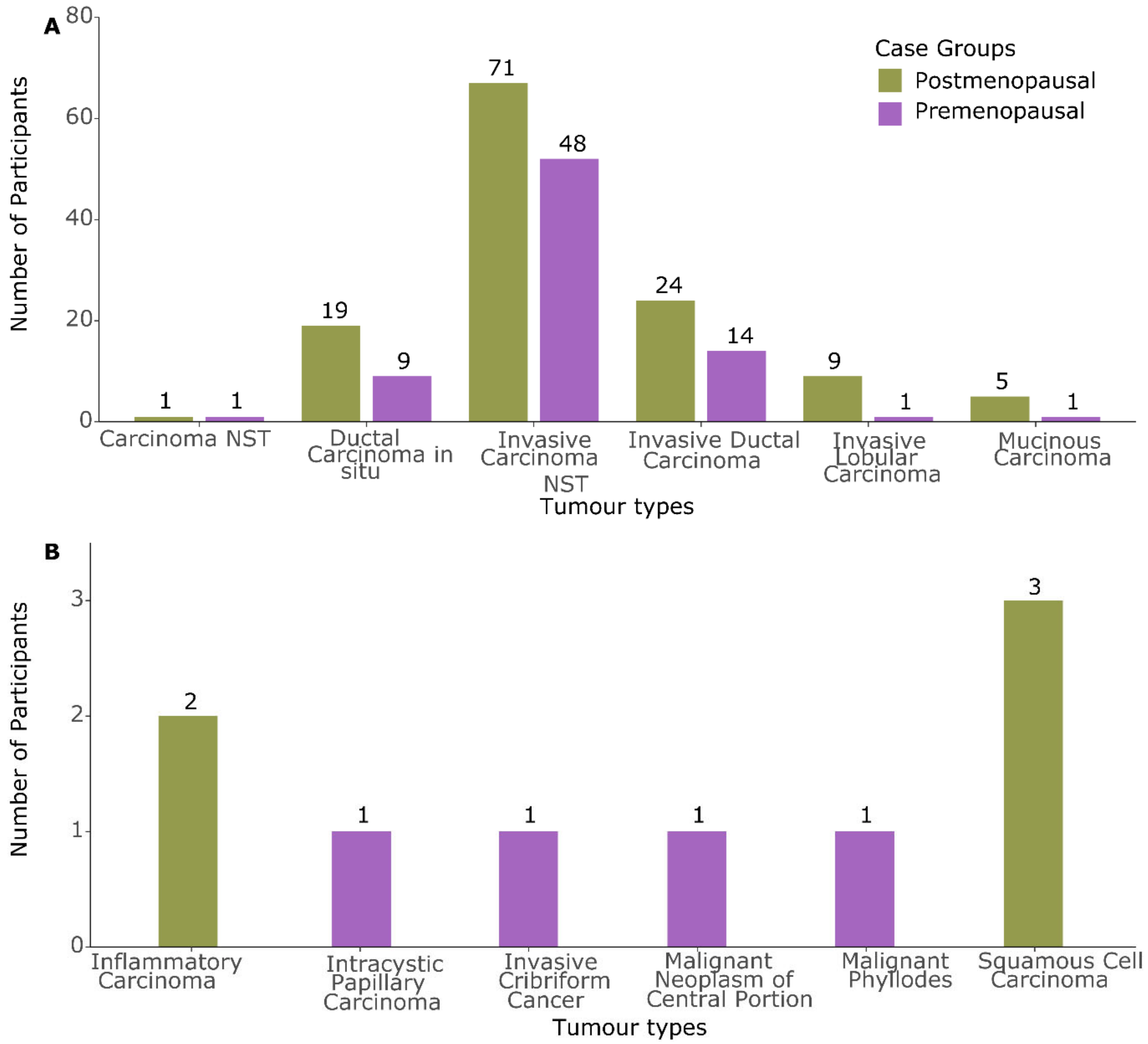

Figure S4: Breast cancer subtypes in pre and postmenopausal breast cancer cases. Tumour types present in both groups (A) and exclusive to each group (B). Invasive carcinoma NST was the most common subtype in both groups, followed by Invasive ductal carcinoma and then ductal carcinoma *in situ*. NST – No special type

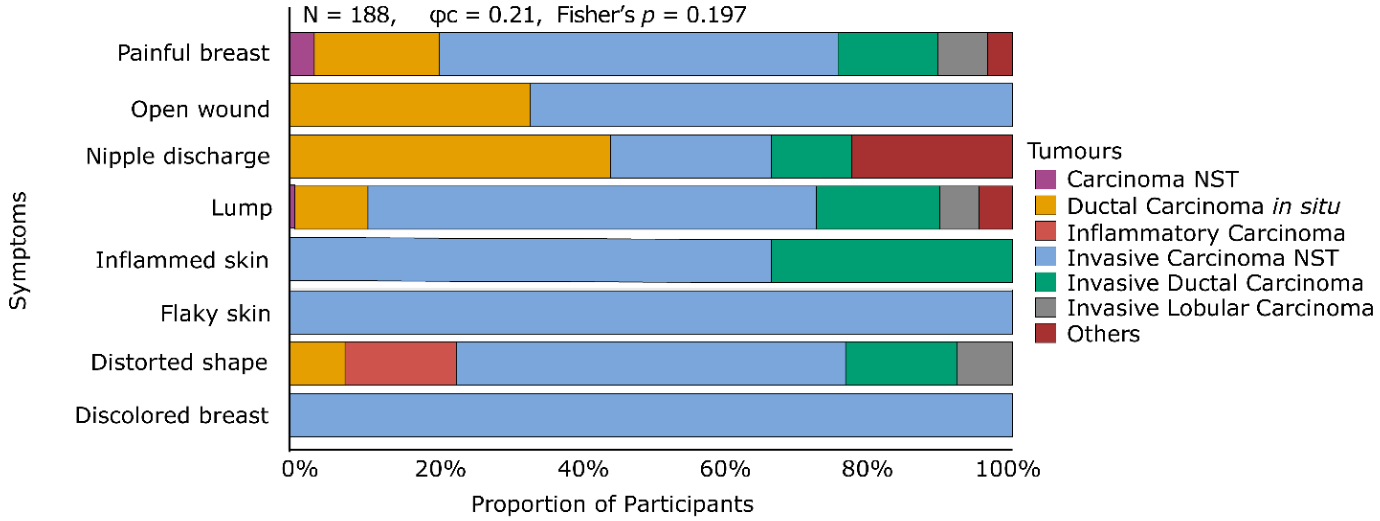

Figure S5: Correlation between the tumor subtypes and symptoms. There was no correlation between the tumor type and symptoms. Invasive Carcinoma NST was the commonest type, irrespective of the symptoms reported. DCIS – ductal carcinoma *in situ*; NST – No special type

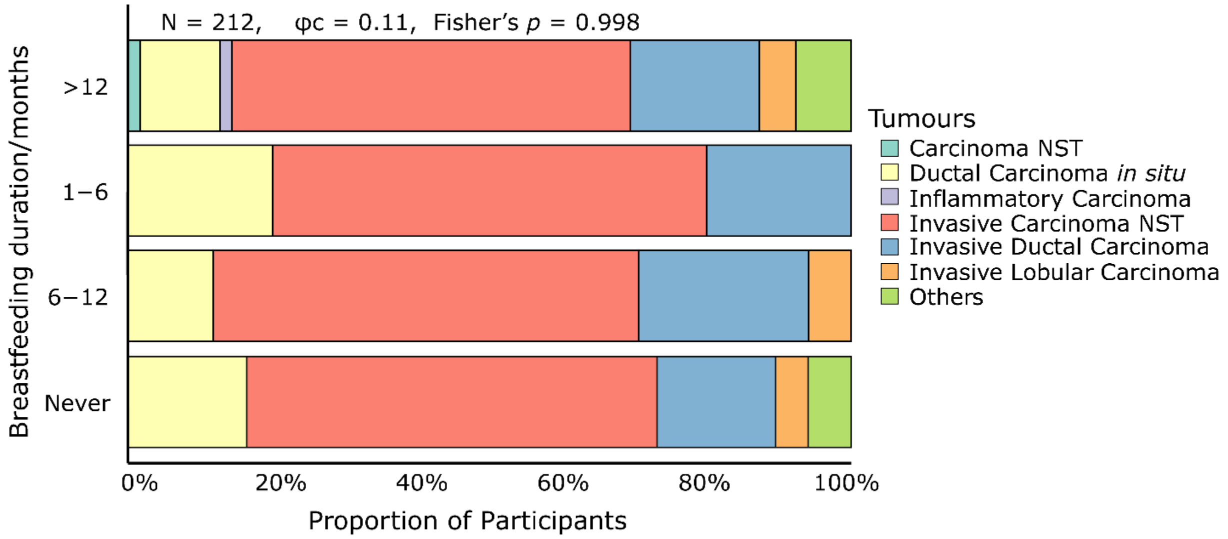

Figure S6: Correlation between Breastfeeding duration and tumor subtypes. There was no correlation between breastfeeding duration and the tumor type diagnosed. Invasive carcinoma NST was the commonest tumor type irrespective of breastfeeding duration. Carcinoma NST and inflammatory carcinoma were only detected in women who breastfed for >12 months

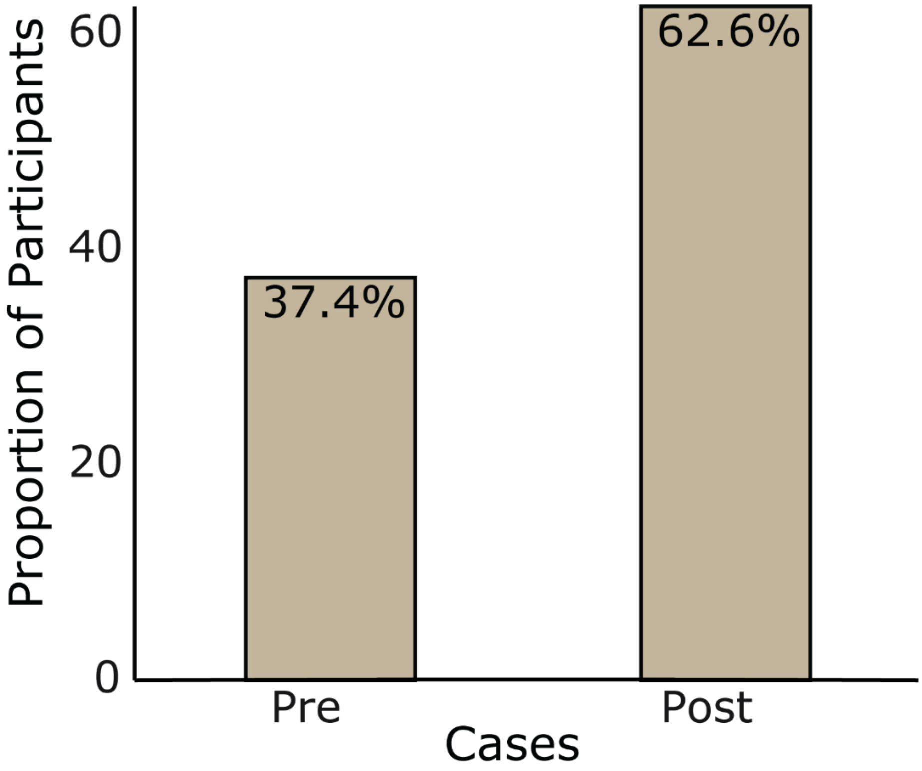

Figure S7: Proportion of pre- and postmenopausal breast cancer categorized using 50 years. Pre – premenopausal; Post – postmenopausal

Table S1: Regional origin of the Ethnolinguistic groups of the Participants

Ethnolinguistic group	*Region of Origin*	*Proportion of Participants (%)*
Dagarti	Upper West	0.4
Akan	Ashanti, Western, Central	44.7
Ga-Adangbe	Greater Accra	24.4
Ewe	Volta	21.5
Sisala	Upper West,	0.4
Gonja	Savannah	0.8
Konkomba	Northern, Brong Ahafo	0.8
Zambraba	"Non-specific"	0.4
Banda Prada	Bono	0.4
Basare	Oti	0.8
Dagomba	Northern	3.7
Mamprusi	Northern	0.8
Kusasi	Northern	0.4
Waala	Upper West	0.8

The Akan group comprised the Fante, Twi and Nzema. Zambraba is an immigrant ethnic group not associated with any distinct region in Ghana.

Table S2: Logistic regression

	Premenopausal			EOBC		
Characteristic	log(OR)1	95% CI	p-value	log(OR)1	95% CI	p-value
Primiparity	0.10***	0.05, 0.15	<0.001	0.09	-0.01, 0.20	0.090
Age at menarche	-0.20**	-0.36, -0.06	0.009	-0.28	-0.57, -0.01	0.051
Menopausal age	-0.24***	-0.38, -0.12	<0.001			
Gravidity	-0.33***	-0.49, -0.18	<0.001	-0.25	-0.66, 0.09	0.195
Parity	-0.41***	-0.61, -0.24	<0.001			

[1]*p<0.05; **p<0.01; ***p<0.001
Abbreviations: CI = Confidznce Interval, OR = Odds Ratio

Table S3: Univariate analysis

	**Premenopausal**			**EOBC**		
**Predictor**	**OR**	**95% CI**	**p-value**	**OR**	**95% CI**	**p-value**
Menarche						
**15**						
<15	1.16	0.56 - 2.39	0.687	1.14	0.47 – 2.73	0.769
>15	0.41	0.18 – 0.92	0.03	0.12	0.25 – 0.56	0.008
Parity						
3						
<3	1.19	0.62 – 2.31	0.598	2.05	0.79 – 5.35	0.142
>3	0.57	0.28 – 1.17	0.129	0.36	0.009 – 1.44	0.148
Never	5.78	2.02 – 16.54	0.001	14.67	4.67 – 46.03	0.0001
Gravidity						
4						
<4	1.62	0.80 – 3.28	0.181	2.34	0.80 – 6.84	0.121
>4	0.63	0.29 – 1.37	0.242	0.58	0.15 – 2.27	0.434
Never	1.85	0.82 – 4.18	0.138	5.48	1.80 – 16.62	0.003
Occupation						
Others						
Student/Unemployed/Retired	0.64	0.02 – 0.18	0.0001	0.12	0.03 – 0.42	0.001
Trader/Businesswoman	0.57	0.31 – 1.04	0.067	0.47	0.22 – 0.97	0.042
Primiparity						
24						
<24	1.76	0.37 – 8.41	0.476			
>24	4.55	0.97 – 21.31	0.055			
Contraceptive use						
No						
Yes				2.78	1.33 – 5.78	0.006

OR = Odds Ratio, CI = Confidence Interval
